# Polyhydroxy-3-Butyrate (PHB)-Based Composite Materials Reinforced with Cellulosic Fibers, Obtained from Barley Waste Straw, to Produce Pieces for Agriculture Applications: Production, Characterization and Scale-Up Analysis

**DOI:** 10.3390/ma17081901

**Published:** 2024-04-19

**Authors:** Helena Oliver-Ortega, Philippe Evon, Francesc Xavier Espinach, Christine Raynaud, José Alberto Méndez

**Affiliations:** 1 Department of Materials Science and Engineering, Universitat Politècnica de Catalunya, Colom 1, 08222 Terrassa, Spain; helena.oliver@upc.edu; 2 Institut d’Investigació Tèxtil i Cooperació Industrial de Terrassa (INTEXTER), Colom 15, 08222 Terrassa, Spain; 3 Laboratoire de Chimie Agro-Industrielle (LCA), Université de Toulouse, ENSIACET (École Nationale Supérieure des Ingénieurs en Arts Chimiques et Technologique), INRAE, Toulouse INP, 4 Allée Emile Monso, 31030 Toulouse Cedex 4, France; philippe.evon@toulouse-inp.fr; 4 LEPAMAP-PRODIS Group, Department of Chemical Engineering, University of Girona, C/M. Aurèlia Capmany, 61, 17003 Girona, Spain; francisco.espinach@udg.edu; 5 Centre d’Application et de Traitement des Agroressources (CATAR), Toulouse-INP, ENSIACET, 4 Allée Emile Monso, 31030 Toulouse Cedex 4, France; christine.raynaud@toulouse-inp.fr

**Keywords:** biocomposite, twin-screw extrusion, injection, computational modelling

## Abstract

Cellulosic fibers obtained from Barley straw were utilized to reinforce PHB. Four different processed fibers were employed as reinforcing material: sawdust (SW), defibered (DFBF), delignified (DBF), and bleached (BBF) fibers. The composite was processed from two different perspectives: a discontinuous (bach) and an intensification process (extrusion). Once processed and transformed into final shape specimens, the materials were characterized by mechanical testing (tensile mode), scanning electron microscopy, and theoretical simulations by finite elements analysis (FEA). In terms of mechanical properties, only the elastic moduli (Et) exhibited results ranging from 37% to 170%, depending on the reinforcement composition. Conversely, strengths at break, under both tensile and bending tests, tended to decrease, indicating poor affinity between the components. Due to the mechanical treatment applied on the fiber, DFBF emerged as the most promising filler, with mechanical properties closest to those of neat PHB. DFBF-based composites were subsequently produced through process intensification using a twin-screw extruder, and molded into flowerpots. Mechanical results showed almost identical properties between the discontinuous and intensification processes. The suitability of the material for agriculture flowerpots was demonstrated through finite analysis simulation (FEA), which revealed that the maximum von Mises stresses (5.38 × 10^5^ N/m^2^) and deformations (0.048 mm) were well below the limits of the composite materials.

## 1. Introduction

European plastic production in 2021 reached 57.2 Mt, with 87.6% comprising fossil-based polymers, 10.1% comprising recycled polymers, also sourced from fossils, and only 2.3% consisting of biobased polymers. France and Spain rank as the third and fourth largest plastic consumers in Europe, accounting for 16.9% of European production [1]. Although the utilization of plastic materials in agriculture and gardening is comparatively low (3.1%) in contrast to sectors like packaging (39.1%), plastic materials in agriculture are primarily single-use due to degradation under usage conditions, rendering them unsuitable for reuse and appropriate recycling. The collection of such materials can also pose challenges, thereby exacerbating the widespread presence and dispersion of plastic materials in the environment [2]. Nevertheless, played a crucial role in advancing agriculture and are currently indispensable in the field.

Biodegradable and biobased materials are increasingly replacing conventional plastic materials, particularly in applications such as packaging and present a promising alternative in the agricultural sector [3]. Some bioplastic materials are already commercially available for short-term agricultural applications. It is due to their biodegradable behavior that decreases rapidly their dimensional stability [4,5]. One of such materials is polyhydroxyalkanoates (PHAs), with polyhydroxy-3-butyrate (PHB) being the most extensively studied variant [6,7]. PHAs, derived from bacteria sources, are biopolyesters that readily biodegrade. Nonetheless, PHAs, particularly PHB, remains costly due to the need for optimization in production methods [8,9]. Furthermore, although PHB exhibits competitive properties compared to polyethylene (PE) and polypropylene (PP), its susceptibility to degradation significantly compromises its stability [10]. To prolong the lifespan of PHB-based products while retaining their biodegradability and reducing material costs, researchers have investigated the reinforcement of this polymer with natural fibers [11,12].

**Figure 1:**
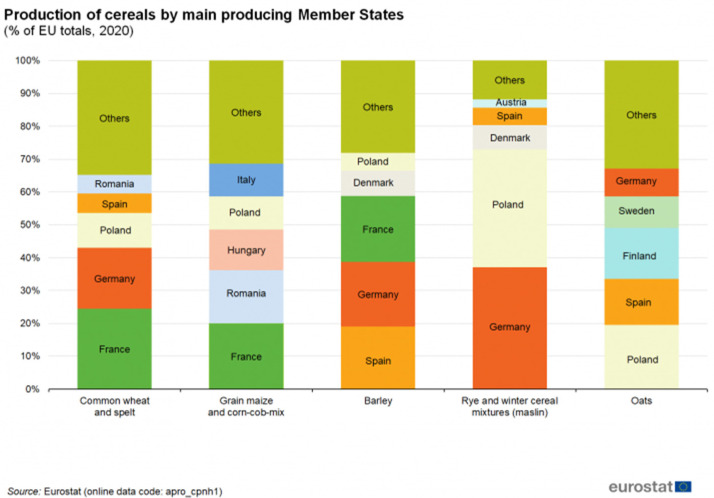
Comparison of the production of different cereals in the EU (Source: Eurostat).

Cellulose and lignocellulosic fibers are commonly used as reinforcements in green polymer composites [13]. Despite having lower mechanical properties compared to glass fibers and carbon fibers, this limitation can be mitigated by increasing fiber content. Moreover, their renewable and biodegradable character make them promising green reinforcements [14]. Additionally, the lower stiffness and brittleness of these reinforcements facilitate their recyclability in composite materials, resulting in reduced damage to processing equipment. Therefore, reinforcing PHB with lignocellulosic fibers could potentially reduce material costs while extending product lifespan without compromising PHB’s biodegradable properties [15]. However, the effectiveness of fiber reinforcement depends on their ability to interact with the polymer matrix. PHB, being a linear polyester, exhibits limited interaction with cellulose, similar to PLA-based reinforced composites [16]. The properties of cellulose, the primary compound in lignocellulosic fibers, including molecular weight, arrangement, and crystallinity, vary depending on the natural resources, highlighting the importance of selection and treatment. Wood and filament fibers are known to offer superior mechanical properties, enhancing reinforcement in polymer composites, but at a high price. Agriculture residues present a greener and cost-effective alternative to these fibers. France and Spain, being major barley producers in the European Union (Figure 1), generate significant quantities of barley straw as a by-product [17]. In this contest, the BIOPLAST European project explores the use of barley straw as a mechanical reinforcement, aiming to produce fully biobased and biodegradable composite materials for agriculture [18]. Utilizing straw as a reinforcement not only reduces production costs but also adds value to agricultural by-products, thereby enhancing crop sustainability.

In this study, the production and characterization of PHB-based composite materials loaded with barley straw will be analyzed, along with their performance in an agricultural product. Barley agricultural residue will undergo various physical and chemical treatments to assess fiber wettability with the PHB matrix. As previously mentioned, the interaction between cellulose and the polymer matrix is crucial and is influenced by the type of fiber used [13,19]. Barley straw contains lower lignin content compared to wood fibers but possesses a higher concentration of extractives, including minerals, waxes, fatty acids, proteins, free sugars, or flavonoids. While these components may enhance fiber dispersion in the PHB matrix, they could diminish stress transmission by inhibiting the interaction with cellulose, the reinforcing agent, as observed in other polyester systems [16]. To assess the feasibility of these composite materials, they will be manufactured using both batch and continuous processes, followed by an economic analysis. Finally, the performance of an injection-molded product, such as a flowerpot, will be simulated and compared to those made from conventional plastics to demonstrate the suitability of these PHB-based as alternatives to petroleum-based materials.

## 2. Materials and Methods

### 2.1. Materials

Polyhydroxy-3-butyrate (PHB) Biomer, grade P209, supplied by Biomer (Schwalbach, Germany) was used as the polymer matrix for formulating the composites. Barley straws were kindly provided by farmers from the region bordering Spain and France.

### 2.2. Fiber Preparation

**Figure 2:**
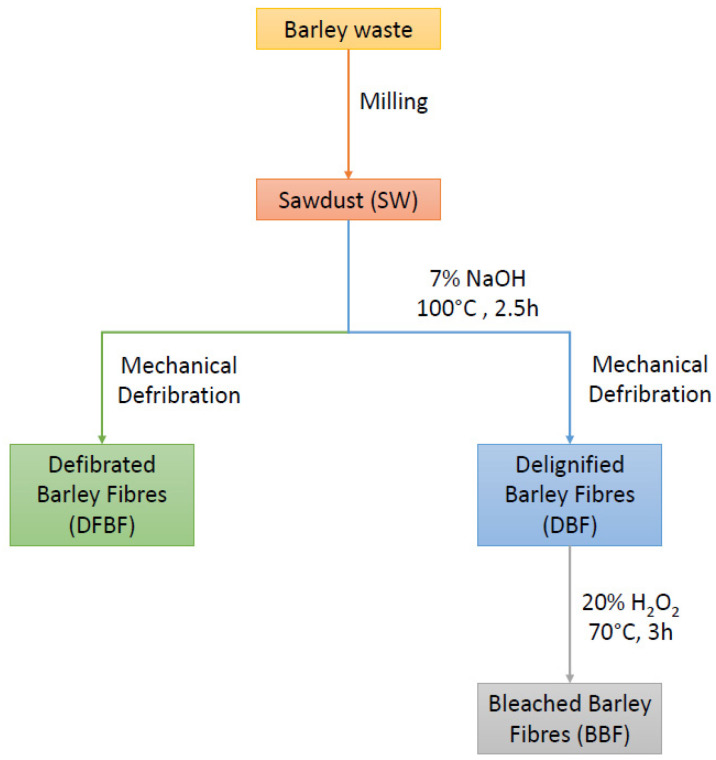
Fiber production processes.

Four different processed fibers were utilized as reinforcement for the composite materials to evaluate the impact of the chemical composition and morphology on composite properties: sawdust (SW); defibrated (DFBF); delignified (DBF); and bleached (BBF) fibers. The different treatments were conducted to assess the effects of fiber chemical composition and morphology on the mechanical performance of the composites. Barley straw was initially cleaned in cold water, dried, and ground through a 5 mm sieve to produce the sawdust (SW). For producing defibrated fibers (DFBF), the sawdust was processed using a Sprout-Waldron mill (Sprout Waldron, Muncy, PA, USA) to accurately separate the fibers. This mechanical process defibrates the barley fibrous residue, yielding individualized fibers without altering the chemical composition of the barley straw, as observed in other cold-water-assisted mechanical processes [20]. Delignified fibers (DBF) were obtained by ambient digestion of the sawdust in a reactor at 100 °C for 2.5 h with NaOH. The NaOH concentration was 7% (*w*/*w* of fibers), and the cooking process consistency was maintained at 10% [21,22]. Subsequently, the fibers were rinsed with distilled water until neutral pH was achieved, and then processed through the Sprout-Waldron mill to obtain individualized fibers. Finally, bleached fibers (BBF) were derived from the DBF delignified fibers. These fibers were submitted to a bleaching process with hydrogen peroxide (H_2_O_2_) [23]. The treatment was conducted at 70 °C, with a consistency of 3% for 3 h. The H_2_O_2_ concentration was 20% (*w*/*w* of fibers), and it was added every 40 min until the 3 h duration was reached. The production of the different reinforcements is summarized in Figure 2.

### 2.3. Fiber Chemical Composition and Morphological Analysis

The chemical composition of the fibers was analyzed following the TAPPI standards: ashes (T211), extractives (T204), and soluble lignin (T222). The holocellulose content, including cellulose and hemicelluloses, was determined by the difference between the total content and the other components.

The morphological analysis of the fibers was carried out using a MorFi fiber analyzer (Techpap, Grenoble, France) with an aqueous suspension of fibers at a concentration of 25 mg/L. Optical microscopy images were captured at ×20 magnification using a laboratory microscope.

### 2.4. Composite Compounding and Samples Obtaining (Discontinuous Production)

PHB-based composite materials with the different reinforcing fibers were prepared using a Gelimat kinetic mixer (Draiswerke, Mahwah, NJ, USA). The reinforcement contents for each formulation were 10, 20 and 30%. Prior to blend production, fibers and PHB were dried in a ventilated oven at 80 °C. The fibers and PHB matrix were then slowly added in the mixing chamber of the Gelimat at low speed (300 rpm). Subsequently, the chamber was closed, and the mixing rate was increased to 2500 rpm. Once the polymer matrix was thoroughly blended with the barley fibers, the chamber was opened, and the obtained material was cooled down and ground for its subsequent transformation. Specimens for the mechanical testing were prepared by injection molding using an Allrounder-220M injection molding equipment (Arburg, Eschweiler, Germany). A temperature profile of 160–165–163–160 °C was established for the transformation. The pressure in the volumetric phase ranged from 300 to 400 bar for the composites with higher fiber content, while it remained at 37 bar during the pressure phase.

### 2.5. Process Intensification

PHB/SW and PHB/DFBF composite materials (90/10 (*w*/*w*) and 70/30 (*w*/*w*) ratios) were also prepared in a continuous production using a Clextral Evolum 25 twin-screw extruder (Firminy, France) having a 1200 rpm maximal screw rotation speed (S_max_). Initially, SW and DFBF were dried overnight at 80 °C, with a moisture content of 3.5% during compounding. Ten successive modules composed the extruder barrel with a total length (L) of 1 m, and a L/D ratio of 40, with D (25 mm) representing the screw diameter. A Schenck Process ProFlex C 500 gravimetric feeder (Darmstadt, Germany) was used for feeding PHB in the first module. A Coperion K-Tron K-CL KT20 volumetric feeder (Stuttgart, Germany) was used for dosing SW and DFBF. They were then conveyed using a side feeder at the fifth module.

The screw profile used was as follows:

- Melting of PHB occurred in the fourth module using two series of bilobe paddles and a series of reverse screw elements, with all three joined together.
- Dispersion of fibers within the molten PHB matrix was achieved through three successive zones of bilobe paddles in modules 6 to 8. The second kneading block was completed by reverse screw elements immediately after.
- Degassing was implemented at the level of the ninth module.

To minimize PHB thermal degradation, a temperature profile was established as follows (from modules 1 to 10, and at the die): 160–170–175–180–175–175–170–165–160–155–155 °C (die). Screw rotation speed (SS, rpm) and total feed rate (Q, kg/h) were set at 200 rpm and 10 kg/h, respectively. A die with two 3 mm diameter holes was positioned. After cooling in a water-cooled channel, the two rods were granulated using a Maag Automatik GmbH PRI-MO 120 E device (Großostheim, Germany).

Specific mechanical energy (SME) at the moment of twin-screw compounding was calculated according to Equation (1).

(1) $$\mathrm{SME}=\frac{U\times I\times \surd 3\times T\times \frac{S_{S}}{S_{\max}}\times \cos\varphi }{Q}$$

where:

U is the motor’s operating voltage [400 V], I the current feeding the motor [57 A], T the motor torque during sampling [%], and cosφ the motor’s theoretical yield [cosφ = 0.90].

Test specimens were prepared from the PHB/SW and PHB/DFBF composite materials, by injection molding using a Negri Bossi VE 160-720 press (Cologno Monzese, Italy). Flowerpots were also manufactured from composite materials containing 30% (*w*/*w*) fibers, as an example of real product. Two different molds were employed: a two-cavity mold for simultaneous production of one tensile specimen and one flexural specimen, and a one-cavity mold for producing one flowerpot per cycle. The temperature profile along the plasticizing screw was 150–160–180 °C, while the nozzle temperature was maintained at 140 °C. Screw speed and counter pressure were set at 100 rpm and 5 bar, respectively. The injection speed and follow-up pressure were 150 mm/s and 700 bar, respectively. Clamping force remained at 1600 kN with a mold temperature of 30 °C and a cooling duration of 30 s.

### 2.6. Mechanical Testing

PHB-based composites produced through discontinuous mixing and injection were subjected to tensile, flexural, and impact tests. Tensile and flexural properties were evaluated according to ASTM D638 [24] and ASTM D790 [25] standard specifications, respectively, using a DTC-10 universal testing machine supplied by IDMtest (Donostia-San Sebastian, Gipuzkoa, Spain) equipped with a load cell of 5 kN. Impact resistance was determined using Charpy methodology in accordance with ISO 179 [26] standard. Samples were tested using a Resil 5.5 impactometer supplied by Ceast (Turín, Italy). Prior to mechanical testing, all the samples were conditioned in a climatic chamber at 23 °C and 50% relative humidity for 48 h. Each mechanical test was conducted on a minimum of 5 specimens.

The PHB/SW and PHB/DFBF composite materials produced using the twin-screw extruder were also subjected to tensile and flexural testing following the same standards. Testing was performed using an Instron 33R 4204 system (Norwood, MA, USA) equipped with a 5 kN load cell. Prior to testing, the samples were conditioned at 25 °C and 60% relative humidity until reaching a constant weight.

### 2.7. Scanning Electron Microscopy (SEM)

Micrographs of the fractured surface of tensile samples were captured using scanning electron microscopy (SEM). The equipment used was a Zeiss DSM 960A microscope (Carl Zeiss Iberia, Madrid, Spain). Prior observation, the samples were coated with gold to enhance conductivity.

### 2.8. Flowerpot Simulation

**Figure 3:**
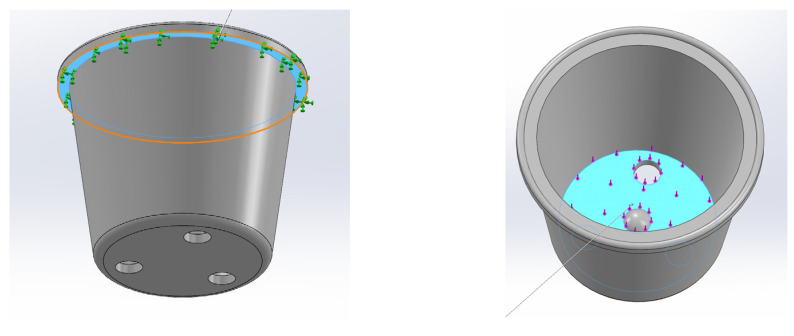
Geometry, contour restrictions, and loads applied to perform the finite element analysis. The restrictions and loads are applied to blue-colored zones. Green arrows indicate a movement restriction. Magenta arrows indicate a load and its direction.

A flowerpot simulation was conducted to assess the mechanical performance of the PHB composites and evaluate the potential as replacements for conventional materials. The flowerpot mock-up was built using Solidworks CAD software (Solidworks Student Edition 2022–2023) developed by Dassault Systèmes (Vélizy-Villacoublay, France) based on the dimensions of a flowerpot manufactured in our facilities. Subsequently, the simulation module of SolidWorks^®^ was used to conduct a finite element analysis (FEA). The study is static. In this analysis, the movement of the body was restricted at the top border and a load of 3 kg was applied to the top of the model (Figure 3).

The model was meshed using 8-node hexahedral elements. The mesh was refined until it was considered satisfactory. The meshing resulted in a total of 30,495 nodes and 17,166 elements. Only 3% of the elements exhibited an aspect ratio below 3, and none were distorted.

## 3. Results and Discussion

### 3.1. Fiber Characterization

The chemical composition of the different fibers utilized as reinforcement are presented in Table 1. The chemical compositions of SW and DFBF fibers are identical due to the Sprout-Waldron equipment mill which does not alter the chemical composition (no removal of any chemical compound occurs during this pre-treatment). Standard deviations are included within brackets.

The chemical composition of untreated fibers (SW and DFBF) was found to be consistent with previously reported compositions [27]. Differences in ashes and extractives may arise from variations in soil composition and growth duration. Consequently, SW and DFBF exhibited a reduced cellulose content on the surface, which could reduce the reinforcement performance of these fibers. Alkaline treatment resulted in the removal of lignin, extractives, and ashes from the DBF fibers. The removal of these components resulted in the increase in the holocellulose content from 71.3% to 80.5%. Additionally, the aggressive character of the treatment likely reduced hemicellulose content, leading to a higher cellulose content. The effect of that treatment is mainly observed on the surface of the fibers, with ashes and extractives being the most impacted fractions and primary surface components. However, lignin remained relatively high compared to the initial composition (i.e., 15.1% instead of 19.6% in SW). A bleaching process was performed to reduce lignin content in BBF fibers. The fibers became whiter, and the lignin content was significantly reduced (down to 10.5%), while ashes and extractives were slightly reduced.

Morphological analysis of the DFBF, DBF and BBF fibers was conducted using the MorFi analyzer. SW fibers could not be analyzed due to the large size of the sawdust particles (5 mm in diameter). The morphological results are shown in Table 2.

**Figure 4:**
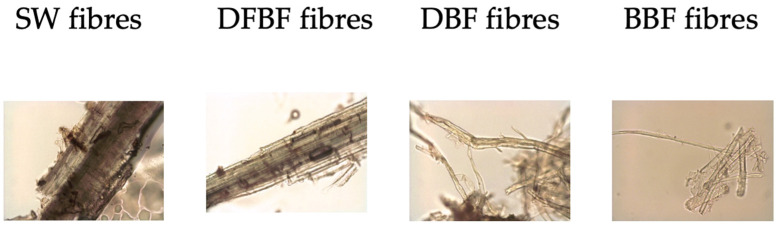
Optical microscope photographs of the reinforcing fibers (×20 magnification).

The alkaline treatment resulted in a noticeable reduction in fiber diameter, attributed to the removal of surface components. This effect is also readily apparent in optical microscope images (Figure 4). SW fibers exhibit a plant-like structure, while the other fibers display a typical fibrous morphology. DFBF fibers do not completely show individualized fibers as observed in DBF and BBF fibers, but the fibrous appearance is clearly observed. Surface fibrillation is observed during this process and is maintained thereafter. The bleaching process slightly reduced the fiber length. Fines, defined are fibers with a length below 75 µm, showed a slightly increase from DFBF to DBF fibers, likely stemming from the alkaline treatment. In the case of BBF fibers, the fines were reduced, possibly due to the loss of part of them during the filtering process.

Agglomerations are clearly visible for DBF and BBF fibers under the optical microscope. This phenomenon is attributed to hornification, which occurs during the drying of the fibers [28]. Consequently, re-dispersing the fibers in water for MorFi analysis becomes challenging. These agglomerations may also inhibit fiber dispersion within the composite materials during the compounding phase, potentially leading to a decrease in their mechanical performance.

### 3.2. Mechanical Performance of the Composites

Tensile and flexural strengths (σ_t_ and σ_f_, respectively), elastic moduli (E_t_ and E_f_), deformations (ε_t_ and ε_f_), and impact resistances, both for unnotched and notched specimens (Iu and In, respectively), of the composites, are shown in Table 3.

**Figure 5:**
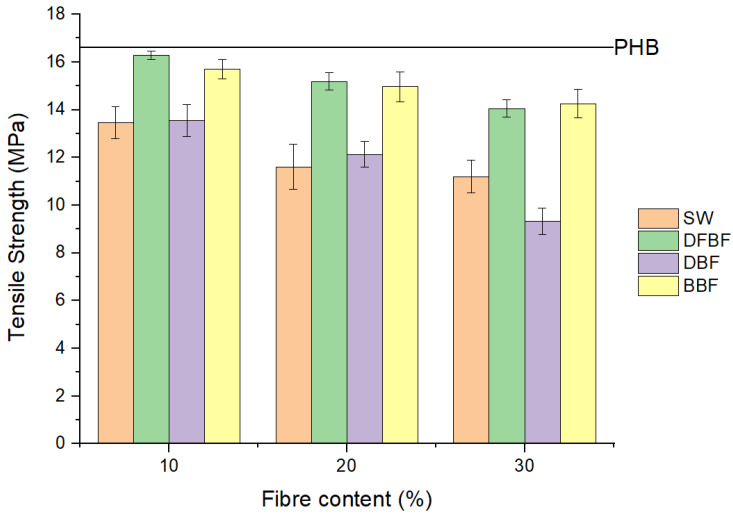
Comparison of the tensile strength of PHB-based composites regarding the fiber used and its content.

No reinforcing effect was observed in the tensile strength of the composites, which is typically one of the main objectives in preparing composite materials. The addition of fibers to the PHB matrix produced a detrimental effect, which was increased with the fiber content (Figure 5). Nonetheless, DFBF and BBF fibers only contributed to a 15% and 14% decrease in tensile resistance, respectively, in 30% filled composites. The stress–strain curves of the materials belong to typical fragile materials with a clear elastic zone followed by a small shoulder before breaking. A similar effect was also observed for the flexural strength of the composites, although in that test, a reinforcement effect of the fibers was obtained with DFBF and BBF fibers. This difference in behavior could be attributed to the combination of the tensile and compression stresses during the flexural test. Moreover, the flexural strength decreased when the fiber content exceeded 20%. High fiber contents could dead to fiber agglomeration, resulting in poor dispersion within the composite material. Additionally, the behavior of strength properties is generally influenced by interactions between the different phases of the composite material. A maximum 21% increment in PHB flexural strength was observed for the PHB-DFBF composite material filled with 20% DFBF fibers (material referenced as PHB-DFBF-20). The small increments observed in some cases and the loss of properties in others indicate limited interaction between barley fibers and the PHB matrix with chemically untreated fibers. The performance of bleached fibers (BBF) was comparable to DFBF, likely due to direct interaction between the fiber and cellulose, the reinforcing component in natural fibers. The results of SW-reinforced composites could be attributed to the low aspect ratio of these fibers, resulting in reinforcement more like particles than fibers, which have a lesser reinforcing effect. Nonetheless, the results of the DBF-reinforced composites were lower than expected, possibly due to fiber agglomeration during compounding, even if lignin removed during delignification process was expected to positively affect interaction with the PHB polymer matrix.

Although the composite materials did not exhibit improved tensile strength compared to neat PHB, their cost will be reduced, as PHB is the most expensive component. Moreover, as mentioned earlier, the strength reduction is minimal for DFBF and BBF fibers. However, the negative effect of the fibers’ presence could enhance the durability of the materials in long-term applications, as PHB degrades and biodegrades easily [29,30].

The behavior of the elastic moduli, both in tensile and in bending, aligns with what was previously observed with the strengths. The addition of fibers in the polymeric phase stiffened the composite material in a linear trend. The best results were obtained for the DFBF fibers, except for the flexural modulus of the PHB-DFBF composite material filled with 30% DFBF fibers (referenced as PHB-DFBF-30), indicating that better dispersion was achieved with these fibers up to a 20% fiber loading. Conversely, this dispersion should have been improved at 30% fiber loading to continue increasing the flexural modulus. SW fibers exhibited high dispersion, likely due to their particle size. DBF- and BBF-reinforced composites attained lower moduli, due to the lower dispersion of such fibers and the presence of aggregates generated during fiber drying.

**Figure 6:**
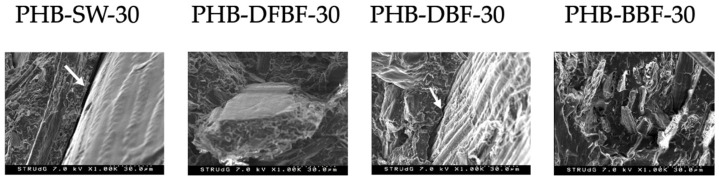
SEM microphotographies at 30 µm of the 30% filled composite materials. Arrows show the interface area between the PHB matrix and fibre.

Deformation is reduced in all composite materials, due to the addition of a stiffened phase, following the same trend observed for the tensile and flexural. Better interactions were observed with the DFBF fibers and BBF fibers. These interactions are evident in the SEM images taken from the tensile-tested samples (Figure 6). SEM images of the SW and DBF-based composites revealed clear voids between the fibers and the polymer matrix (identified in the images with white arrows). Moreover, in the case of the DBF fibers, their diameter appears to exceed 30 µm, illustrating the agglomeration phenomenon resulting from the poor dispersion as reported in the mechanical properties study [31]. DFBF fibers exhibited a larger diameter, also found in the MorFi analyzer (Morfi 2010). Nonetheless, a better wettability of these fibers was demonstrated. Finally, BBF-reinforced material showed broken fibers but also some voids at the fracture level, indicating fiber slipping during testing.

Impact resistance is a crucial property for applications where sudden impact occurs rather than a continuous applied stress. It is likely one of the most common stresses in real conditions for agricultural pots, films, covers, etc. The addition of the fibers to the PHB matrix resulted in a significant reduction in impact resistance in both notched and unnotched samples (Table 3). This reduction is magnified by adding more fibers to the composite material as the interphase volume, representing the most fragile phase in the material consisting of interactions between fibers and the polymer matrix, becomes increasingly significant.

The resistance of composite materials to impact is generally related with the energy absorbed by the matrix and fibers (W_m_ and W_f_, respectively), the energy required to initiate the fracture (W_i_), and the energy absorbed by the interphase (W_fm_) (Equation (2)). The energy required to initiate a fracture can be estimated from the difference between the impact resistance values of the unnotched and notched samples.

(2) $$W\;\approx {\;W}_{i}+W_{f}+W_{m}+\sum W_{\mathrm{fm}}$$

**Figure 7:**
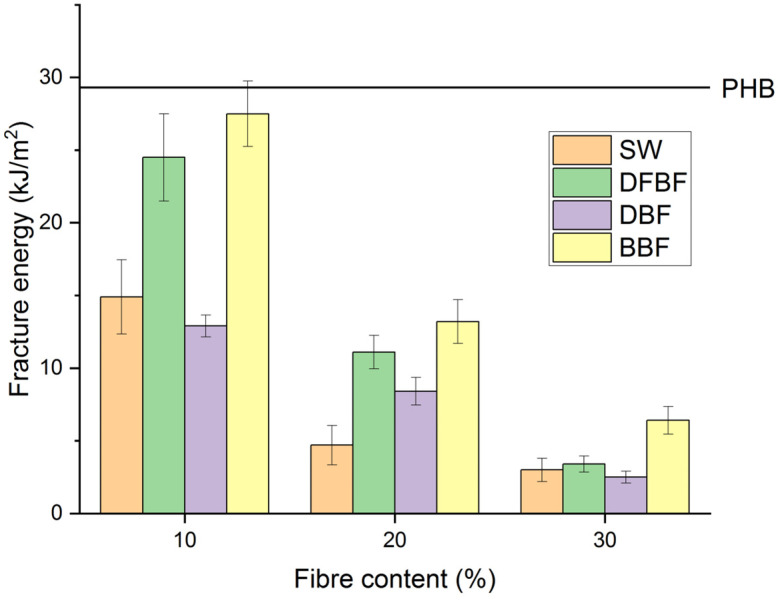
Fracture energy for the PHB-based composite materials.

Figure 7 illustrates the fracture energies calculated for all the composite materials. Except for the formulations reinforced with 10% fiber content of DFBF and BBF fibers, all the composites exhibited a loss of fracture energy greater than 50% compared to neat PHB, indicating that fracture in the material will be easily. The PHB-BBF-10 composite material demonstrated the highest resistance to fracture, while the PHB-DFBF-10 exhibited similar energy. However, higher fiber content drastically reduced this energy. For PHB-DFBF composites, the results are attributed to better wettability of the fibers, indicating a better interface as observed in the SEM images. Conversely, the results obtained with composites made of bleached fibers are unexpected, as the different polarities may lead to lower energetic interactions. Nonetheless, energy absorbed and dissipated by cellulose could be higher than that by lignocellulosic fibers, where lignin covers the fiber surface and is not covalent-bonded to cellulose chains, thus dissipating less energy.

### 3.3. Fiber Chosen for Process Intensification: Performance and Economical Evaluation

**Figure 8:**
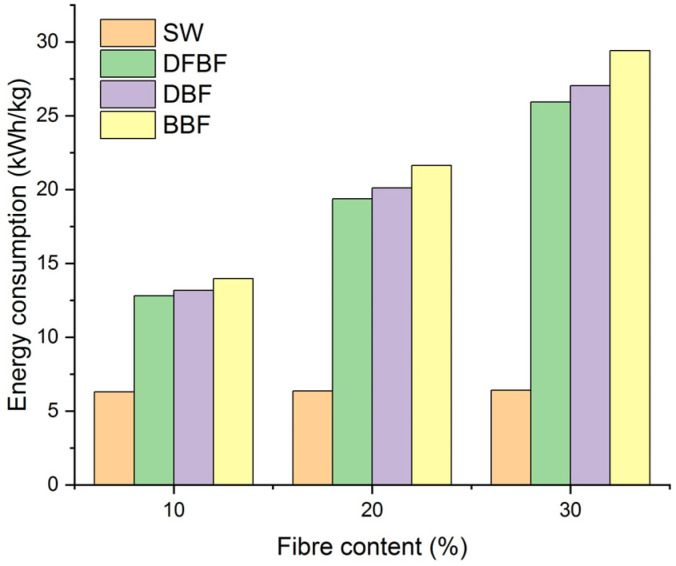
Energy consumption per kg of composite material produced.

The study of the mechanical performance of composite materials in a discontinuous process led to the proposal of using defibrated (DFBF) and bleached (BBF) fibers as reinforcing fibers. These fibers have demonstrated superior performance in the composite materials. However, the manufacture processes of these two fibers are entirely different, with the production of bleached fibers involving two additional steps compared to the defibrated fibers. These additional steps result in higher energy consumption and the generation of wastewater. Therefore, an analysis of the energy and reactants consumptions is warranted. PHB is currently an expensive polymer matrix. While the addition of fibers could potentially reduce costs by decreasing the proportion of the more expensive phase, if the fibers themselves are costly to produce, it could lead to an undesirable increase in price. The energy consumption of the composites prepared at the laboratory scale is shown in Figure 8.

**Figure 9:**
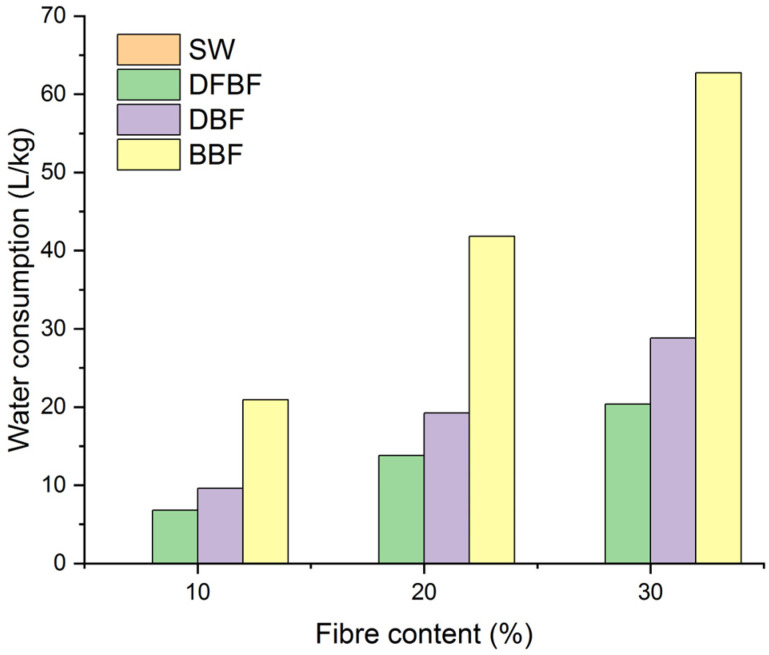
Water consumption per kg of composite material produced.

The energy consumption includes the milling, drying, compounding and transformation processes, along with additional steps for the fiber production. The most costly aspect was the defibration process, exhibiting a notable increase in the energy consumption for DFBF. This energy usage was slightly increased for the DBF and BBF fibers. Regarding water consumption (Figure 9), the Gelimat mixer accounted for the majority of its consumption for the SW-based composites. However, this water could potentially be recycled with a closed system and thus not factored into the calculation. Water consumption increased again in the composite materials when the Sprout-Waldron equipment was employed for the defibration process. Moreover, there was also a significant additional increase in water consumption for the BBF fibers due to the cooking, bleaching, and washing stages.

**Figure 10:**
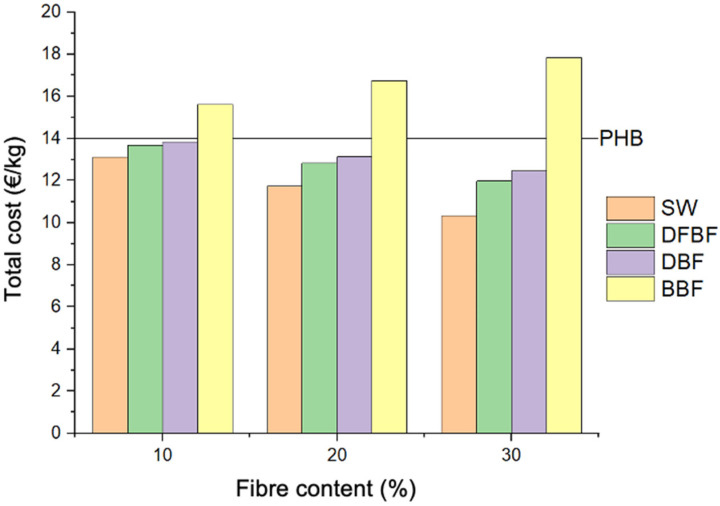
Total production cost per kg of composite material produced.

Furthermore, the pricing of the composites heavily relies on the chemical reagents (NaOH and H_2_O_2_) and the cost of PHB matrix. Spanish electrical energy costs (0.083 EUR/kW·h in a mixture consumption in 2019) and the industrial water supply costs in Girona (Spain) (0.653 EUR/m^3^) were utilized for the calculation (Figure 10). The incorporation of reagents resulted in a substantial increment in the composite costs, surpassing those of neat PHB for all the composites produced with chemically treated fibers.

Despite the superior reinforcing effect of BBF compared to the other materials produced in this work, the production cost of BBF-based composites exceeded that of neat PHB. Opting for more economical barley fibers while enhancing the dimensional stability of the PHB-based composites over time appears to be a preferable choice. In that regard, process intensification was conducted with the SW- and DFBF-based composites.

### 3.4. Process Intensification of the Composite Compounding through Twin-Screw Extrusion

For process intensification, composite compounding was carried out continuously in a twin-screw extruder. The raw materials utilized as reinforcement were sawdust (SW) and defibered fibers (DFBF), as mentioned previously. Two filler contents were tested, i.e., 10% (*w*/*w*) and 30% (*w*/*w*), respectively. The extrusion process was also applied to neat PHB as control. Subsequently, granules were molded into test specimens to characterize both PHB and the composite materials in terms of mechanical performance.

Thanks to the three mixing zones along modules 6 to 8, aggregates within DFBF were well disintegrated, facilitating efficient dispersion of both types of reinforcing fibers within PHB matrix. This resulted in a stable compounding process, illustrated by stable motor torques throughout production (Table 4). Furthermore, with the addition of more fibers, the increase in specific mechanical energy (SME) was quite slight (+15% max), meaning that fibers were well dispersed, even when larger quantities were added. With SME values ranging from 190 to 219 W h/kg, and using the 0.083 EUR/kW·h electrical energy cost in Spain, this resulted in a production cost for twin-screw extrusion compounding that comprised between 0.016 and 0.018 EUR/kg granules (cost excluding the extruder’s amortization), which remains very low in comparison with the cost of PHB (generally situated between 8 and 10 EUR/kg).

In terms of scalability, although the total feed rate was only 10 kg/h in the present study, it is reasonable to assume that a feed rate of at least 50 kg/h could be achieved by working at the maximum extruder’s screw rotation speed (i.e., 1200 rpm) rather than 200 rpm as practiced here. In addition, twin-screw technology can easily be scaled up. For example, the entire Evolum range of Clextral machines has been designed to allow direct volumetric extrapolation thanks to a homothetic design. As an example, an Evolum 53 extruder (53 mm for the screw diameter) has a free volume almost ten times greater than that of the Evolum 25 model used here. Such a machine, considered as an entry-level industrial machine, could reasonably produce granules at around 500 kg/h. It remains to be seen whether the dispersion of the fibers in the bioplastic matrix will be as good as ever at such production rates. If this is not the case, it may be necessary to adapt the screw and/or temperature profiles.

Injection of the test specimens from the produced granules was possible without any difficulty in automatic mode, even at the highest filler content, which perfectly illustrates the robustness of this shaping method for a wide range of applications. From a visual point of view, the injected specimens made from DFBF appeared slightly clearer and more homogeneous than those made from SW. Table 5 and Table 6 summarize the tensile and bending properties of the injected materials.

When comparing the tensile properties of neat PHB (Table 3) and extruded PHB (Table 5), a slight degradation of PHB has been observed due to the extrusion process. This was evidenced by a slight decrease in the tensile strength (from 16.6 MPa to 15.3 MPa) and especially by a significant reduction in elongation at break (from 13.3% to 5.3%). This suggests that the operating conditions at the moment of compounding could be adapted in the future, for example by slightly reducing the temperature profile in the mixing zone, where the fibers are dispersed in the molten PHB matrix. Adding SW and DFBF reduces PHB tensile strength, due to a lack of chemical affinity between the matrix and the filler. Moreover, for composite materials made from the extruded granules, the tensile strength remained in the same order of magnitude as for the composites produced using the discontinuous process (Table 3), even at 30% filler content, thus evidencing an effective dispersion of SW and DFBF fibers during extrusion compounding, which is however a short residence time process (around 2 min). The 10% and 30% filled DFBF-based extruded materials also revealed higher tensile strength than the SW-based ones. The same was observed with the Gelimat mixer, thus evidencing a more favorable morphology of the defibered fibers in comparison with the large size of SW particles.

The main differences between composites made with the discontinuous process and those originating from extrusion were observed for Young’s moduli and elongations at break. Although PHB stiffening was still observed with extruded materials (Table 5), the increase in the Young’s modulus in the presence of fibers was less significant compared with the Gelimat materials. In the same way, reduction in the PHB elongation at break was still effective inside the extruded materials. However, it was less significant than for the Gelimat ones. This suggests that the reinforcing fibers were probably reduced in length during the extrusion compounding process, although this could not be confirmed experimentally. For this purpose, image analysis of the fibers contained in the injected parts after prior dissolution of the PHB matrix, for example using chlorinated solvents such as chloroform or dichloromethane, would have been necessary. In the future, a slightly less mechanically shearing screw profile would probably make it possible to limit this phenomenon of fiber length reduction.

The flexural properties of the materials from the extruded granules are summarized in Table 6. The addition of fibers to PHB resulted also in more rigid materials in bending. However, higher values of elongation at break were preserved for the extruded composites, even with a 30% (*w*/*w*) filler content, in comparison with the Gelimat ones. Lastly, when comparing flexural strengths at break of extruded materials each other, those of the DFBF-based ones were significantly higher. This confirms once again that the defibration process applied to sawdust to produce DFBF improved the fiber morphology, enabling better adhesion between PHB and DFBF.

**Figure 11:**
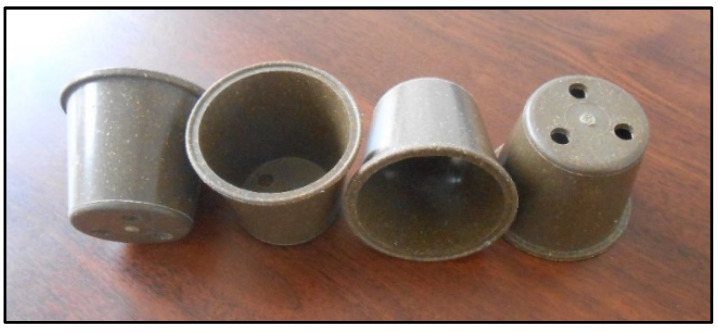
Example of flowerpot injected from the PHB-DFBF-30 composite material.

Granules made of 30% (*w*/*w*) SW or DFBF were also used to mold flowerpots by injection (Figure 11). Injection was still possible in automatic mode despite the more complex shape of the pots compared with standard tensile and bending specimens. The mold cavity was always perfectly filled, which can be explained by the low melt rheology of the biocomposite mixture, even at this highest filler ratio. The future industrialization of injection molding will require multi-cavity molds. This will boost the productivity of the forming process. Nevertheless, injection molding is known as a high-capacity shaping technique, robust in industry, that should pose no major obstacles, given the initial trials already successfully completed. The next paragraph will be dedicated to the simulation of the mechanical performance of these flowerpots.

### 3.5. Mechanical Assessment of Composite Materials: Flowerpots Simulation

**Figure 12:**
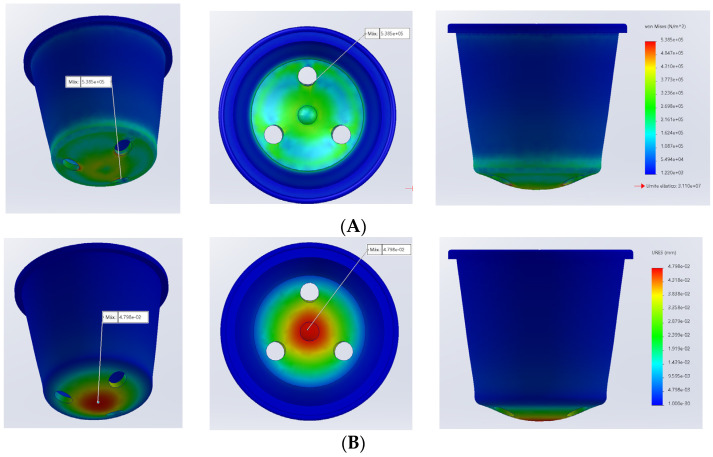

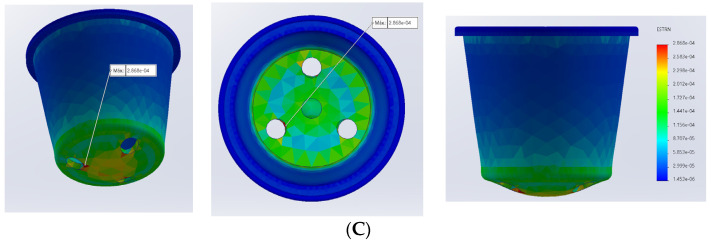
Results of FEA for PHB ((**A**) von Mises stress distribution; (**B**) total deformation; (**C**) single-element strain).

The finite element analyses provided information regarding load distribution within the model, the deformations, and the strains as well as a security factor. This analysis was conducted for all the materials listed in Table 5. The load distribution, represented as von Mises loads, did not exhibit significant changes among materials because it is more related to the loads, movement restrictions, and model geometry. However, the deformation did change because it is related to Young’s moduli of the materials. Figure 12 shows the results obtained for PHB.

The maximum von Mises stress is located near the bottom holes of the flowerpot. It can be expected that this maximum stress coincides with the zone submitted to the highest deformation, but the hole acts as a stress amplifier, potentially leading to some flattening phenomena in these areas. The elements submitted to the maximum unitary strains are also located in these zones. In the case of PHB, the safety factor was 30. This is a substantial value, indicating that the thickness of the flowerpot walls could be reduced.

Table 7 shows the results obtained for neat PHB, for all the composite materials analyzed (materials made of SW and DFBF fibers, added at 10–30% (*w*/*w*) fiber loading), and for the common plastics typically used for flowerpots, i.e., high-density polyethylene (HDPE) and polypropylene (PP).

The obtained results for all the composite materials were lower than the observed ones for the common plastics and neat PHB. Nonetheless, it was expected that HDPE, PP, and PHB resistances were higher than those of the PHB-based composite materials, whatever the fiber studied and its filling rate. Moreover, maximum deformations in composite materials were lower, due to the higher stiffness in these materials, which was the consequence of the PHB reinforcement with cellulosic fibers.

Finally, the safety factor indicates the suitability of the product to be used as a flowerpot in real conditions. A higher value than 1 is necessary for the material to be considered for such a use. All the PHB-based materials, including neat PHB, exhibited values lower than that observed for PP. However, all safety factors showed values much higher than 1, indicating the possibilities to optimize the design of the flowerpot. This is interesting as the amount of material is directly correlated to the environmental impact of the flowerpot: the more the amount of material, the more the impact of the flowerpot for the environment. The authors performed FEA in the case of a flowerpot with a 1 mm wall thickness. This product, with a 77% reduction in mass, revealed a safety factor of 4.4 and a maximum deformation of 1.6 mm. Nevertheless, it will be necessary for future work to practically evaluate the ability of these composite materials to properly fill such a mold cavity.

One of the objectives is to incorporate fiber contents as high as possible in a composite material, resulting in a reduction in the polymer volume. PHB is currently a relatively expensive biobased polymer as its isolation process after biological synthesis by bacteria remains costly. Including high contents of fibers reduces the cost of the final product, although the price remains expensive in comparison with common plastics. Thus, it is helpful to reduce the cost in fiber preparation as much as possible. BBF are relatively expensive fibers, considering both time and required reactants. Moreover, their yield is around 70% (*w*/*w*), representing a significant loss of material. This is the reason why the DFBF fibers, which were only mechanically treated, were identified in the present study as the barley fibers preferred as a filler for PHB, being a good compromise between production cost and ability for PHB mechanical reinforcement.

## 4. Conclusions

A study of the production, properties, intensification production, and mechanical assessment of composite materials composed of PHB and fibers obtained from barley straw has been developed. Fiber treatment of barley reinforcement revealed a null reinforcement effect in terms of tensile strength, but certain composites improved the flexural resistance (i.e., those based on DFBF and BBF fibers). The elastic modulus was increased for all the composites. Process intensification was carried out with the DFBF fibers, i.e., the most easily scalable fibrous material that also showed one of the best performances in the composites. In the discontinuous process, samples did not exhibite a reinforcement effect in mechanical terms, likely due to the poor affinity between PHB and fibers. The material with 30% DFBF was successfully injected in automatic mode, demonstrating the capability for easy large-scale production. Finally, the mechanical assessment of flowerpots was studied through simulation, with results indicating the suitability of the produced materials to replace common petrol-based products.

## Figures and Tables

**Table 1:** Chemical composition of reinforcing fibers in % of dry matter. Standard deviation is described between brackets.

	SW/DFBF Fibers	DBF Fibers	BBF Fibers
Ashes [%]	6.8 (0.4)	2.7 (0.1)	2.1 (0.7)
Extractives [%]	2.3 (0.3)	1.7 (0.3)	1.4 (0.2)
Lignin [%]	19.6 (0.2)	15.1 (2.2)	10.5 (3.5)
Holocellulose [%]	71.3	80.5	86.0

**Table 2:** Morphological data of fibers.

Fiber	Mean Weighted Length [µm]	Diameter [µm]	Fines [%]
DFBF	291.6 (24.8)	30.5 (1.2)	82.7 (1.4)
DBF	290.0 (32.6)	21.8 (0.4)	90.9 (7.5)
BBF	279.5 (0.7)	21.7 (0.3)	74.6 (14.4)

Standard deviations are reported between brackets.

**Table 3:** Mechanical properties of PHB and PHB-based composites.

Sample	Fiber Content [%]	Tensile Properties			Flexural Properties			Impact Resistance	
		σ_t_ [MPa]	E_t_ [GPa]	ε_t_ [%]	σ_f_ [MPa]	E_f_ [GPa]	ε_f_ [%]	Iu [kJ/m^2^]	In [kJ/m^2^]
PHB	0	16.6 (0.2)	0.80 (0.01)	13.3 (1.1)	23.1 (1.1)	0.31 (0.20)	8.3 (0.2)	41.6 (1.9)	12.0 (1.6)
PHB-SW	10	13.5 (0.7)	1.10 (0.05)	3.7 (0.3)	22.3 (2.8)	1.25 (0.11)	4.4 (0.7)	18.8 (3.8)	3.9 (1.3)
	20	11.6 (0.9)	1.54 (0.05)	1.6 (0.5)	22.8 (2.1)	1.78 (0.09)	3.0 (1)	8.2 (2.3)	3.5 (0.4)
	30	11.2 (0.7)	1.85 (0.05)	1.1 (0.1)	22.1 (0.6)	2.62 (0.09)	1.5 (0)	6.8 (1.3)	3.8 (0.3)
PHB-DFBF	10	16.3 (0.2)	1.12 (0.04)	5.1 (0.1)	27.2 (0.3)	1.45 (0.07)	6.0 (0.3)	29.7 (5.7)	5.2 (0.3)
	20	15.2 (0.3)	1.61 (0.03)	2.8 (0.1)	27.9 (0.5)	2.10 (0.06)	3.8 (0.4)	15.7 (1.9)	4.6 (0.4)
	30	14.1 (0.4)	2.17 (0.13)	1.3 (0.1)	22.6 (0.2)	1.41 (0.07)	1.5 (0.1)	6.1 (0.8)	2.7 (0.3)
PHB-DBF	10	13.6 (0.7)	1.38 (0.05)	2.2 (0.3)	23.2 (0.4)	1.50 (0.03)	3.0 (0.2)	16.4 (1.1)	3.5 (0.4)
	20	12.1 (0.5)	1.64 (0.03)	1.3 (0.1)	20.6 (0.8)	1.93 (0.05)	1.6 (0.1)	12.1 (1.8)	3.7 (0.1)
	30	9.3 (0.6)	1.80 (0.08)	0.7 (0.1)	19.2 (1.6)	2.24 (0.07)	1.1 (0.2)	5.1 (0.3)	2.6 (0.5)
PHB-BBF	10	15.7 (0.4)	1.11 (0.02)	5.4 (0.3)	25.4 (0.8)	1.27 (0.08)	7.0 (0.3)	33.9 (4.2)	6.4 (0.3)
	20	15.0 (0.6)	1.51 (0.05)	2.8 (0.3)	27.0 (0.8)	1.78 (0.04)	4.3 (0.1)	18.1 (2.9)	4.9 (0.1)
	30	14.3 (0.6)	1.85 (0.07)	1.8 (0.1)	24.6 (0.8)	2.03 (0.05)	2.8 (0.2)	10.9 (1.7)	4.5 (0.2)

Standard deviations are reported between brackets.

**Table 4:** T and (SME) values during twin-screw extrusion.

Sample	Filler Type	Filler Content [%, *w*/*w*]	Motor Torque [%]	SME [W·h/kg]
PHB	-	0	32.4 (0.9)	191 (5)
PHB-SW-10	SW	10	31.7 (0.9)	190 (5)
PHB-SW-30	SW	30	37.0 (0.3)	219 (2)
PHB-DFBF-10	DFBF	10	33.2 (0.6)	194 (4)
PHB-DFBF-30	DFBF	30	33.3 (0.8)	197 (5)

Values are expressed as mean values ± standard deviations (in brackets).

**Table 5:** Tensile properties of the composite materials made from the extruded granules.

Sample	Filler Type	Filler Content [%, *w*/*w*]	σ_t_ [MPa]	E_t_ [GPa]	ε_t_ [%]
PHB	-	0	15.3 (0.5)	0.80 (0.03)	5.3 (0.4)
PHB-SW-10	SW	10	14.9 (0.3)	0.97 (0.01)	5.0 (0.4)
PHB-SW-30	SW	30	12.2 (0.3)	1.06 (0.06)	2.5 (0.3)
PHB-DFBF-10	DFBF	10	16.8 (0.8)	0.87 (0.05)	3.8 (0.4)
PHB-DFBF-30	DFBF	30	13.7 (0.4)	0.99 (0.06)	2.5 (0.1)

Values are expressed as mean values ± standard deviations (in brackets).

**Table 6:** Bending properties of the composite materials made from the extruded granules.

Sample	Filler Type	Filler Content [%, *w*/*w*]	σ_f_ [MPa]	E_f_ [MPa]	ε_f_ [%]
PHB	-	0	31.1 (0.4)	0.88 (0.06)	10.2 (0.4)
PHB-SW-10	SW	10	24.7 (1.7)	1.14 (0.10)	5.8 (0.6)
PHB-SW-30	SW	30	25.6 (0.7)	1.64 (0.09)	2.8 (0.3)
PHB-DFBF-10	DFBF	10	28.4 (1.9)	1.37 (0.08)	3.9 (0.5)
PHB-DFBF-30	DFBF	30	28.0 (1.1)	1.89 (0.06)	2.5 (0.1)

Values are expressed as mean values ± standard deviations (in brackets).

**Table 7:** Mechanical assessment of the composite materials versus neat PHB and common plastics.

	HDPE	PP	PHB	SW-Based Composites			DFBF-Based Composites		
Fiber Content (%)	0	0	0	10	20	30	10	20	30
Maximum Von Misses resistance before break (MPa)	22.1	24.5	16.6	13.5	11.6	11.2	16.3	15.2	14.1
Maximum von Mises stress in flowerpot (MPa)	0.5	0.5	0.5	0.5	0.5	0.5	0.5	0.5	0.5
Deformation (10^−2^ mm)	4.7	4.6	4.7	4.4	4.2	4.0	4.8	4.8	4.3
Maximum unitary strain (10^−4^%)	2.8	2.8	1.4	2.6	2.5	2.4	2.9	2.9	2.6
Safety factor	41	45	30	27	21	21	30	28	26

## Data Availability

Data are contained within the article.
